# O-GlcNAcylation: roles and potential therapeutic target for bone pathophysiology

**DOI:** 10.1186/s12964-024-01659-x

**Published:** 2024-05-21

**Authors:** Xiaohan Yan, Jingjing Zheng, Wenhao Ren, Shaoming Li, Shuying Yang, Keqian Zhi, Ling Gao

**Affiliations:** 1 Department of Oral and Maxillofacial Reconstruction, the Affiliated Hospital of Qingdao University, Qingdao, 266555 China; 2 School of Stomatology, Qingdao University, Qingdao, 266003 China; 3 Key Lab of Oral Clinical Medicine, the Affiliated Hospital of Qingdao University, Qingdao, 266003 China; 4 Department of Oral and Maxillofacial Surgery, the Affiliated Hospital of Qingdao University, 1677 Wutaishan Road, Huangdao District, Qingdao, 266555 Shandong China; 5 Department of Endodontics, the Affiliated Hospital of Qingdao University, Qingdao, 266003 China; 6 Department of Basic & Translational Sciences, School of Dental Medicine, University of Pennsylvania, Philadelphia, PA 19104 USA

**Keywords:** Post-translational modification, O-GlcNAcylation, Bone pathophysiology

## Abstract

O-linked N-acetylglucosamine (O-GlcNAc) protein modification (O-GlcNAcylation) is a critical post-translational modification (PTM) of cytoplasmic and nuclear proteins. O-GlcNAcylation levels are regulated by the activity of two enzymes, O-GlcNAc transferase (OGT) and O‑GlcNAcase (OGA). While OGT attaches O-GlcNAc to proteins, OGA removes O-GlcNAc from proteins. Since its discovery, researchers have demonstrated O-GlcNAcylation on thousands of proteins implicated in numerous different biological processes. Moreover, dysregulation of O-GlcNAcylation has been associated with several pathologies, including cancers, ischemia-reperfusion injury, and neurodegenerative diseases. In this review, we focus on progress in our understanding of the role of O-GlcNAcylation in bone pathophysiology, and we discuss the potential molecular mechanisms of O-GlcNAcylation modulation of bone-related diseases. In addition, we explore significant advances in the identification of O-GlcNAcylation-related regulators as potential therapeutic targets, providing novel therapeutic strategies for the treatment of bone-related disorders.

## Introduction

The functional diversity of the proteome is considerably augmented by post-translational modification through glycosylation, phosphorylation, methylation, ubiquitylation, and acetylation. In recent years, the pathophysiological roles of these post-translational modifications (PTMs) have attracted a great deal of attention [1, 2]. Glycosylation, the post-translational modification of proteins with various carbohydrates, is one of the most important classes of PTMs, with approximately half of all proteins in living organisms exhibiting this modification [3]. Glycosylation affects protein function, regulates cell signaling, and modulates a range of biological processes [4–6]. O-linked N-acetylglucosamine protein modification (O-GlcNAcylation) was first identified by Hart and Torres in 1984. These authors used bovine milk galactosyltransferase to couple tritiated UDP-galactose to N-acetylglucosamine (GlcNAc) residues on the surfaces of mouse lymphocytes [7]. Nowadays, after in-depth research, the number of O-GlcNAcylated proteins identified has reached more than 4000 [8].

O-GlcNAcylated proteins are typically localized in the nucleus, cytoplasm, or mitochondria [9, 10]. Extensive research has revealed that O-GlcNAcylated proteins play important roles in a variety of cellular processes, including transcription, translation, apoptosis, the cell cycle, protein transportation, mitochondrial function, and signal transduction [11–13]. Moreover, dysregulation of O-GlcNAcylation has been associated with several pathologies, including cancers [14], ischemia-reperfusion injury [15], and neurodegenerative diseases [16]. However, the precise role of O-GlcNAcylation in bone pathophysiology remains incompletely understood. Here, we focus on the effects of O-GlcNAcylation changes on bone physiology and pathophysiology and discuss its potential value as a target in clinical treatment.

## O-GlcNAcylation and related enzymes

**Fig. 1:**
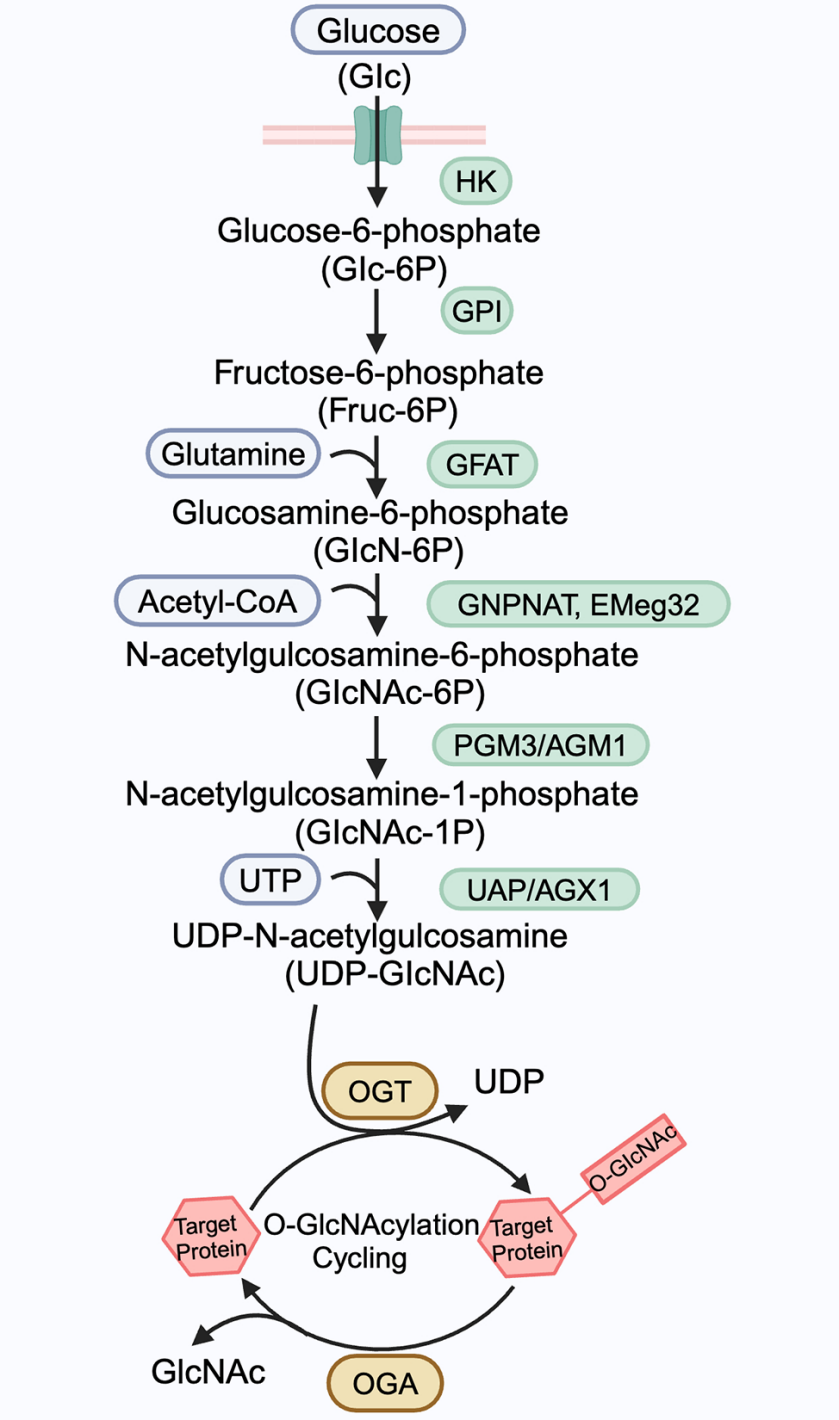
The hexosamine biosynthesis pathway and O-GlcNAcylation The hexosamine biosynthetic pathway (HBP) integrates four metabolic intermediates, a carbohydrate (glucose), an amino acid (glutamine), a lipid (Acetyl-CoA), and a nucleotide (UTP), to produce UDP-N-Acetlyglucosamine (UDP-GlcNAc). O-GlcNAcylation is cycled on and off proteins by two enzymes, O-GlcNAc transferase (OGT) and O-GlcNAcase (OGA), that catalyze the addition and removal of O-GlcNAc, respectively Abbreviations used in this figure: HK, hexokinase; GPI, glucose-6-phosphate isomerase; GFAT, glutamine-fructose-6-phosphate amidotransferase; GNPNAT (EMeg32), glucosamine-phosphate N-acetyltransferase; PGM3/ AGM1, GlcNAc phosphomutase; UAP/ AGX1, UDP-GlcNAc pyrophosphorylase

O-GlcNAcylation functions as a nutrient sensor through the hexosamine biosynthetic pathway (HBP). Because the HBP requires input from several molecules, including glucose, glutamine, UTP, and acetate, it plays a crucial role in sensing and integrating nutrients [17, 18]. Under normal conditions, a fraction (2–3%) of the glucose entering the cell is directed to the HBP [19]. In this pathway, glutamine-fructose-6-phosphate amidotransferase (GFAT) converts fructose 6-phosphate into glucosamine 6-phosphate, and this is ultimately converted into UDP-N-acetylglucosamine (UDP-GlcNAc), which is a key substrate for protein O-GlcNAcylation. The addition and removal steps of UDP-GlcNAc modification are mediated by two highly conserved enzymes, O‑GlcNAc transferase (OGT) and O‑GlcNAcase (OGA), respectively [20](Fig. 1).

**Fig. 2:**
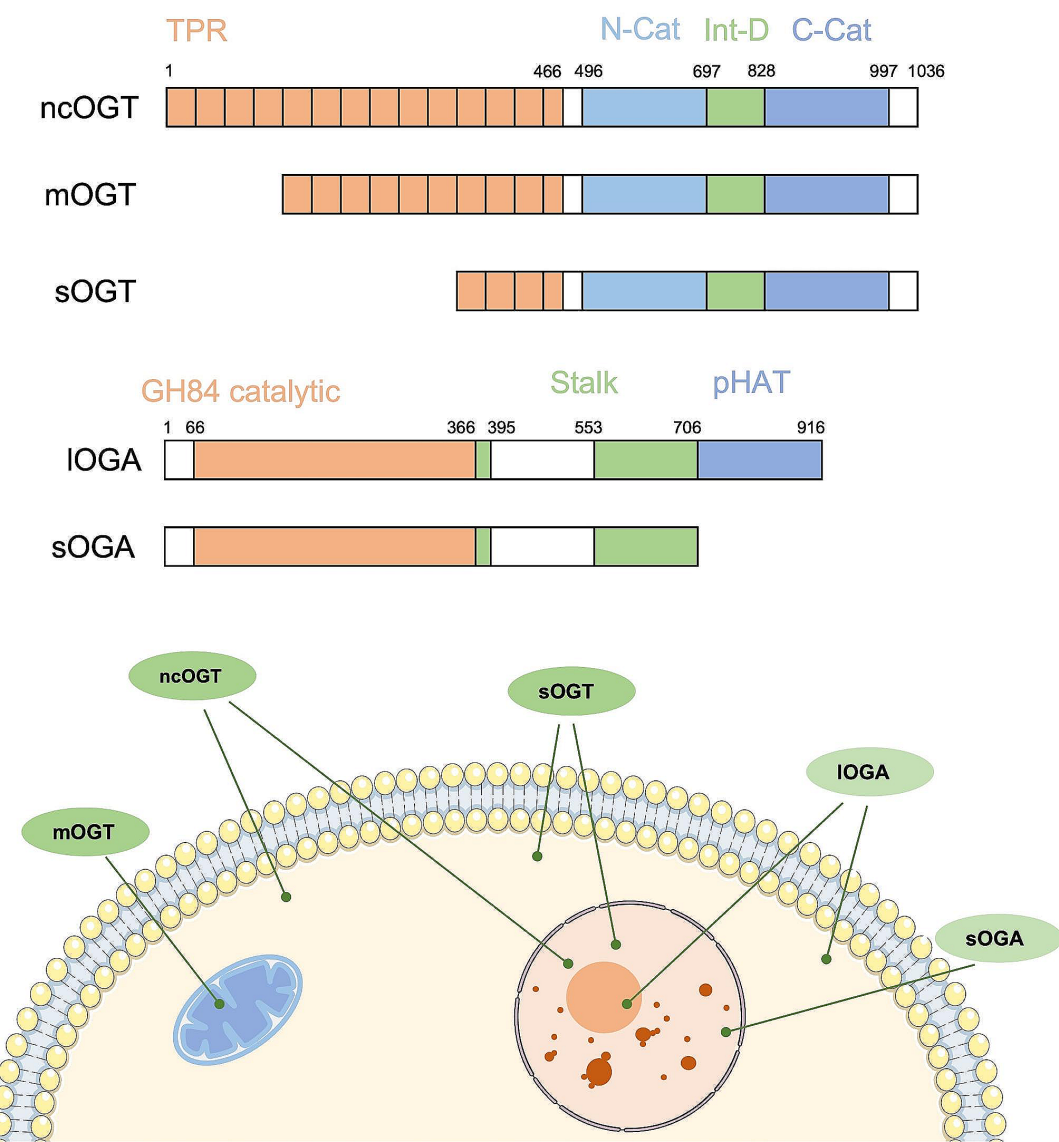
Schematic representation of the OGT and OGA isoforms Schematic representation of the OGT and OGA structural domains, with amino acids labeling the boundaries of each. While ncOGT and sOGT are both distributed in the nucleus and cytoplasm, mOGT is mainly localized in the mitochondria. These isoforms have 13.5, 9.5, and 2.5 (orange) TPRs, respectively. The N-terminal catalytic domain (N-cat) and C-terminal catalytic domain (C-cat) are the two catalytic structural domains (blue), and the intervening domain (Int-D) (green) is of unknown function. lOGA is distributed in the cytoplasm and nucleolus, while sOGA is located in the nucleus. Both isomers contain the same N-terminal catalytic structural domain (Catalytic) (orange) and stalk structural domain (Stalk) (green), but the C-terminal HAT-like structural domain (pHAT) (blue) is absent in sOGA

O-GlcNAc transferase (OGT), which is encoded by a single gene on the X chromosome (*OGT*), was first isolated in 1992 from rat liver and subsequently cloned five years later [21–23]. OGT is highly conserved in mammals, and it is essential for embryonic stem cell viability and development [21, 24]. Functional OGT enzymes are composed of an N-terminal substrate recognition domain and a C-terminal catalytic domain. In the cells tested, three different transcripts are produced by alternative gene splicing, and these transcripts encode a nucleocytoplasmic isoform (ncOGT, 116 kDa), a mitochondrial isoform (mOGT, 103 kDa), and a short isoform (sOGT, 75 kDa). As shown in Fig. 2, the C-termini of these isoforms are identical, although their N-termini differ in the number of the N-terminal tetratricopeptide repeats (TPR) motifs [25]. The unique lengths of their N-terminal domains and differences in the relative positioning of the TPR motifs allow for the targeting of different subsets of protein substrates within the proteome [26]. Through this mechanism, OGT modulates almost every biological process that occurs within the cell.

O‑GlcNAcase (OGA) is the only enzyme capable of hydrolyzing O-GlcNAcylation modifications from various glycoprotein substrates. OGA has two major splice isoforms, long (lOGA) and short (sOGA). Both isoforms are comprised of a glycoside hydrolase 84 (GH84) catalytic domain at the N-terminus and a helical bundle stalk-like domain. However, while lOGA possesses a pseudo-histone acetyltransferase domain (pHAT) in its C‑terminal region, sOGA lacks this pHAT-like domain [17, 27, 28]. The hydrolase activity responsible for removing O-GlcNAcylation is situated in the GH domain (Fig. 2). To date, information concerning the mechanism underlying substrate recognition by OGA is limited. Further elucidation of the structural features of OGA should uncover its substrate recognition mechanism.

In recent years, multiple studies have demonstrated that O-GlcNAcylation plays a crucial role in regulating the dynamic balance of the bone matrix, ultimately constituting a complex network involved in bone metabolism. As a consequence of this important role, O-GlcNAcylation exhibits potential for exploitation as a new molecular marker and as a target for bone therapy.

## O-GlcNAcylation in bone physiology

### The role of O-GlcNAcylation in BMSCs

As an important and dynamic post-translational modification in mammalian cells, O-GlcNAcylation plays a crucial role in the differentiation of cell lineages, such as bone marrow mesenchymal stromal cells (BMSCs), neural stem cells (NSCs) [29], and hematopoietic stem cells (HSCs) [30, 31]. BMSCs are a heterogeneous group of multipotent stem cells, including osteochondral and adipocyte progenitors, that exhibit niche forming and immunomodulatory capabilities [32]. BMSCs are essential for maintaining bone homeostasis, tissue regeneration, and global energy homeostasis. Glucose, fatty acids, and amino acids are known to fuel BMSC differentiation. Plasticity in energy metabolism enables BMSCs to meet the different demands of osteo-adipogenic differentiation. Targeting the metabolic pathways of BMSCs could offer a new therapeutic approach for diseases related to imbalanced BMSC differentiation [33]. Interestingly, researchers have observed changes in intracellular O-GlcNAcylation levels during the differentiation of BMSCs into adipogenic, chondrogenic, and osteogenic cells [34]. In short, O-GlcNAcylation modifies and regulates transcripts, which in turn affects the differentiation fate and niche function of early BMSCs, ultimately coordinating the early development of skeletal and hematopoietic systems.

### The role of O-GlcNAcylation in osteogenic differentiation

Osteoblasts, which are bone-forming cells, play a crucial role in the modeling and remodeling of bones. Osteogenic differentiation is dependent upon multiple signaling pathways, including the Wnt, BMP (bone morphogenetic protein), TGF-β, and hedgehog (Hh) pathways [35]. However, the molecular mechanisms that underlie this process are not completely understood. Runt-related transcription factor 2 (Runx2), a master transcription factor for osteogenesis, orchestrates the differentiation of mesenchymal stem cells into osteoblasts, and these then mature into osteocytes [36]. Researchers have partially elucidated the mechanisms through which O-GlcNAcylation affects the activity of Runx2. Kim et al. first reported that O-GlcNAcylation modification of Runx2 positively regulates its transcriptional activity, resulting in increased expression of its target gene osteocalcin, which may itself indirectly participate in osteoblastic differentiation [37]. Additionally, by modifying and activating Runx2, O-GlcNAcylation promotes osteogenic differentiation of BMSCs, thus controlling niche function [38]. Inhibition of OGA also augmented the transcriptional activity of Runx2, which in turn influenced the production of alkaline phosphatase (ALP), a matrix maturation marker, during the early phases of osteoblast differentiation [39].

As mentioned above, osteogenic differentiation is also dependent upon the Wntpathway. The Wnt signaling pathway can be divided into two main pathways, the canonical pathway, and the non-canonical pathway. The canonical Wnt signaling pathway is mediated by β-catenin, which promotes osteogenesis [40]. A study by Garg et al. demonstrated that treatment with triptolide reduced the expression level of OGT and decreased O-GlcNAcylation modification of β-catenin, thus inhibiting the canonical pathway in pancreatic cancer cells [41]. OGT-mediated O-GlcNAcylation is also known to regulate embryonic neurogenesis and neuronal development in vivo and in vitro by increasing β-catenin levels [42]. Although the mechanism underlying osteogenic differentiation remains unclear, this topic still provides reliable insights for our study.

**Fig. 3:**
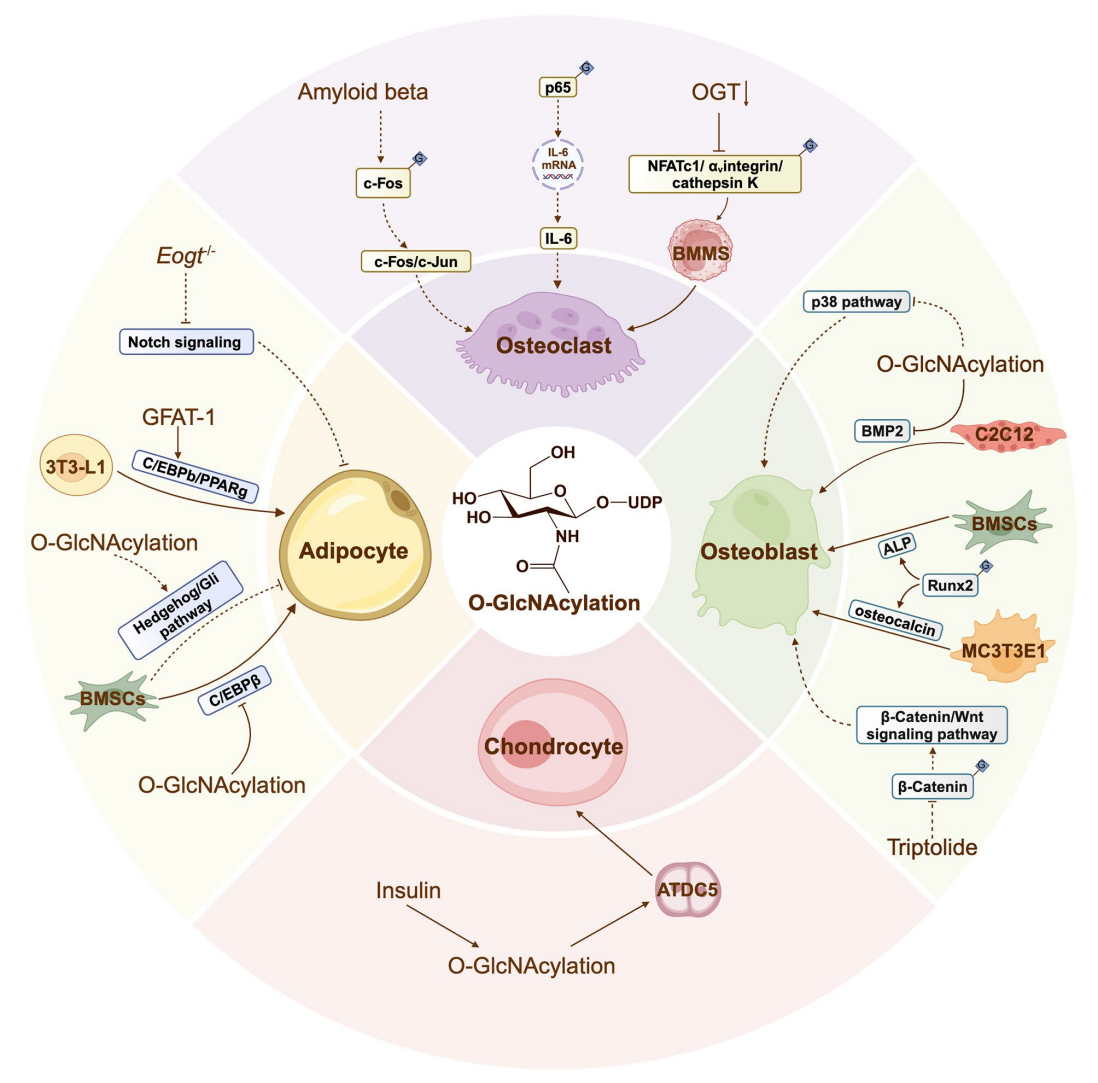
The molecular mechanism and physiological regulation roles of O-GlcNAcylation modification in bone O-GlcNAcylation is involved in osteogenic differentiation, osteoclastic differentiation, adipogenic differentiation, and chondrogenic differentiation, therein regulating the expression of potential target genes and related signaling pathways. Dotted arrows represent a possible but not yet established effect

Interestingly, several studies have reported that O-GlcNAcylation has the opposite effect on osteogenic differentiation, which reveals the contradictory nature of O-GlcNAcylation regulation. For instance, diabetic patients under hyperglycemic conditions may experience an abnormal increase in O-GlcNAcylation. In C2C12 cells, an increase in O-GlcNAcylation was followed by a reduction in bone BMP2-induced osteogenic differentiation [43]. The p38 mitogen-activated protein kinase (MAPK) pathway — one of the three major MAPK signaling pathways, along with the extracellular signal-regulated kinase (ERK) pathway and the c-Jun N-terminal protein kinases (JNK) pathway — is known to be involved in inducing osteogenic differentiation [44]. Papanicolaou et al. found that decreasing O-GlcNAcylation caused activation of basal p38 phosphorylation (and downstream signaling) in the early phase of cardiac myocytes [45]. Similarly, Goldberg et al. [46]. revealed that high levels of O-GlcNAcylation promote cancer progression by downregulation of p38 MAPK activity in the tumor microenvironment (Fig. 3). This opposing effect also occurs in other types of stem cells, such as NSCs [29, 47], which suggests that O-GlcNAcylation has a diverse and multifaceted role in regulating cytogenesis. Hence, O-GlcNAcylation should be considered an important molecular target for the osteogenic differentiation of BMSCs, and further research into its role could offer a new approach to clinical therapy of bone-related diseases.

### The role of O-GlcNAcylation in osteoclastic differentiation

Osteoclasts are tissue-specific macrophage polykaryons that degrade bone matrix, and they are known to differentiate from monocyte/ macrophage precursor cells at or near the bone surface [48]. Recent studies have demonstrated that O-GlcNAcylation is a prerequisite for osteoclast differentiation from these precursor cells, indicating that O-GlcNAcylation also plays a role in the formation of osteoclasts.

Activation of nuclear factor-kappaB (NF-κB) pathways is also required for the maturation and activation of osteoclasts [49]. Moreover, nuclear factor of activated T-cells c1 (NFATc1), a transcription factor, is known to play a crucial role in regulating the expression of osteoclast-specific downstream target genes [50]. Kim’s group found that HBP was activated during the differentiation of murine osteoclasts. Furthermore, OGT deficiency inhibited the O-GlcNAcylation of NF-κB p65 and NFATc1, preventing their transfer to the nucleus and reducing downstream transcriptional activities that control osteoclast differentiation [51]. Similarly, a genetic deletion of OGT inhibited osteoclast formation and downregulated markers related to their differentiation and function, such as α(v) integrin and cathepsin K [52].

Interleukin-6 (IL-6) is a cytokine that regulates B-cell differentiation and was first identified and cloned by Kishimoto and Hirano in 1986 [53, 54]. In a co-culture system comprised of RAW264.7 cells and the human synovial sarcoma cell line SW982, IL-6 enhanced the expression of NFATc1, leading to the induction of osteoclast differentiation [53]. Hu et al. found that the increased O-GlcNAcylation of p65 enhanced its activity and nuclear translocation, leading to the upregulation of IL-6 in the skeletal muscle of mice during exposure to cold temperatures [55]. In addition, amyloid beta (Aβ) is known to increase O-GlcNAcylation of c-Fos, a vital component of activator protein-1. As shown in Fig. 3, O-GlcNAcylation increases the stability of c‐Fos, stimulating both its interaction with c‐Jun and transcriptional activity in neuronal cells [56, 57]. This process may also be essential for osteoclast differentiation [58]. Together, the above findings should guide future research on osteoclastic differentiation of BMSCs, even if not all these mechanisms have been observed in osteoclasts.

### The role of O-GlcNAcylation in adipogenic differentiation

Bone marrow adipocytes are a unique cell population originating from BM mesenchymal progenitors and marrow adipogenic lineage precursors, and they play important physiological roles in hematopoiesis and bone metabolism [59]. As discussed above, O-GlcNAcylation modification is a novel and essential post-transcriptional modulator of gene expression, and several studies have linked it to adipogenic differentiation. Zhang et al. demonstrated that O-GlcNAcylation negatively affects C/EBPβ-dependent marrow adipogenesis [38]. As an early transcription factor, C/EBPβ plays a decisive role in adipogenic differentiation of BMSCs [60, 61]. However, Ishihara et al. found that O-GlcNAcylation was drastically increased during adipogenic differentiation of 3T3-L1 preadipocytes, and that this increase was associated with C/EBPβ expression [62]. GFAT-1 is also known to regulate adipogenesis by increasing the expression levels of critical transcription factors (C/EBPβ, PPARγ), fatty acid synthase (FAS), and lipid droplet (LD) proteins in 3T3-L1 cells [63]. These results suggest that the effects of O-GlcNAcylation are cell-specific and condition-specific. Further studies are needed to elucidate these mechanisms.

Notch signaling is a critical communication pathway between cells that influences numerous cell fate decisions during development [64]. Osathanon et al. found that the Notch pathway demonstrates both inhibitory and stimulatory effects on adipogenic differentiation [65]. Hao et al. provided evidence that the expression levels of Notch signaling-activated genes, *Rbpj* and *Hes1*, were significantly induced in wild-type (*Eogt*^+/+^) CD4^+^ T cells but not in *Eogt* knockout (*Eogt*^-/-^) counterparts. Thus, loss of *Eogt* appears to suppress Notch signaling pathway [66]. The Hedgehog (Hh) family of signaling proteins is known to control differentiation and proliferation during development [67]. During the adipogenic differentiation of BMSCs, Hh signaling is downregulated due to the decreased expression of transcription factor Gli [68]. Interestingly, O-GlcNAcylation modification functionally increased transcriptional activity in the Hh/ Gli pathway in tumor cells (Fig. 3) [69]. Although there is no direct evidence that regulation of adipogenic differentiation by O-GlcNAcylation involves the above mechanisms, these studies provide new directions for further research on adipogenic differentiation.

### The role of O-GlcNAcylation in chondrogenic differentiation

Cao et al. utilized the chondrogenic differentiation of BMSCs for treating and repairing cartilage defects [70]. In fact, some promising in vitro studies have reported the chondrogenic effects of glucosamine (GlcN), which include chondrocyte proliferation [71, 72]. However, the mechanisms regulating O-GlcNAcylation modification during this process are not fully understood.

O-GlcNAcylated protein expression is increased during insulin-induced Pre-chondrogenic ATDC5 cell differentiation, suggesting a possible link between O-GlcNAcylation and chondrocyte differentiation. In addition, accumulation of O-GlcNAcylation after inhibition of OGA leads to pre-hypertrophic chondrocyte differentiation in vitro and in vivo in newborn mice (Fig. 3) [73]. However, our current understanding of this topic is limited, and further research is needed to better understand the role of O-GlcNAcylation in regulating chondrogenic differentiation and development.

## O-GlcNAcylation participates in pathological bone diseases

### Rheumatoid arthritis (RA)

RA is a chronic inflammatory joint disease associated with loss of cartilage, bone damage, and disability [74]. An accumulation of immune cells and expansion of resident stromal and vascular cells in the local pathology generates an aggressive environment that leads to gradual bone mass loss [75]. However, the pathogenic cause of this disease is not yet fully understood. Establishing an early diagnosis, initiating prompt treatment, and designing novel treatment strategies to control inflammation, minimize damage, and prevent further complications are crucial.

Tumor necrosis factor alpha (TNF-α), a key mediator of inflammation in arthritis, fosters the dynamic regulation of O-GlcNAcylation during osteoclastogenesis [76]. Mechanically, TNF-α enhances osteoclastogenesis in inflammatory arthritis by increasing the expression levels of OGT and OGA. Kim et al. found that hyper-O-GlcNAcylation of p65 significantly aggravated the severity of RA in response to TNF-α. This was due to increased nuclear translocation of p65, increased binding of p65 protein to DNA, and enhanced transcriptional activity [77]. The cytokine Interleukin-1β (IL-1β) is involved in the perpetuation of the chronic inflammation state characterizing RA [78]. Umar et al. found that Penta-o-galloyl-beta-d-Glucose (PGG) could inhibit O-GlcNAcylation of transforming growth factor beta-activated kinase 1-binding protein 1 (TAB1) and reduce TGF-β-activated kinase 1 (TAK1) activation in IL-1β-stimulated human RA synovial fibroblasts (RASFs). The resulting reduction in TAK1 signaling led to a reduction in inflammation and tissue destruction [79]. Another critical inflammatory pathway in RA is the NF-kB signaling pathway [80]. Previous studies have shown that O-GlcNAc modification of NF-kB positively regulates its gene transcription functions in different cell types [81–84]. These findings may inform future treatments for RA.

Dendritic cells (DCs) are specialized cells that present antigens, and they play a crucial role in immunity and immune tolerance [85]. In RA, there is a massive infiltration of DCs in the synovium. Monocyte-derived dendritic cells (moDCs) in the RA synovium facilitate the production of pro-inflammatory cytokines [86]. Weiss’s group demonstrated that OGT-mediated O-GlcNAcylation modulates the AKT and MEK/ ERK pathways in moDCs, ultimately hindering the differentiation of monocytes into immature DCs, and impeding the maturation process in already differentiated immature moDCs [87].

Macrophages in the immune system can be divided into two types: classically activated macrophages (M1) and alternatively activated macrophages (M2), which perform pro-inflammatory and anti-inflammatory functions, respectively [88]. In RA, the imbalance between pro-inflammatory M1 and anti-inflammatory M2 macrophages leads to synovial inflammation, autoimmunity, and joint damage [89]. Yang et al. reported that OGT-mediated O-GlcNAcylation modifications suppress macrophage pro-inflammatory polarization by regulating S6 kinase beta 1 (S6K1) O-GlcNAcylation/ phosphorylation and S6K1 activation [90]. Nagai-Singer et al. demonstrated that NOD-like receptor (NLR) X1 (NLRX1) suppresses pro-inflammatory NF-κB signaling and blocks downstream pro-inflammatory factors [91]. Chen et al. found that elevated O-GlcNAcylation levels promoted NLRX1 ubiquitination, which enhanced the binding of NLRX1 and IkappaB kinase-alpha (IKK-α). This ultimately led to a reduction in the expression of the inflammatory cytokine IL-1β in M1 macrophages [92].

**Fig. 4:**
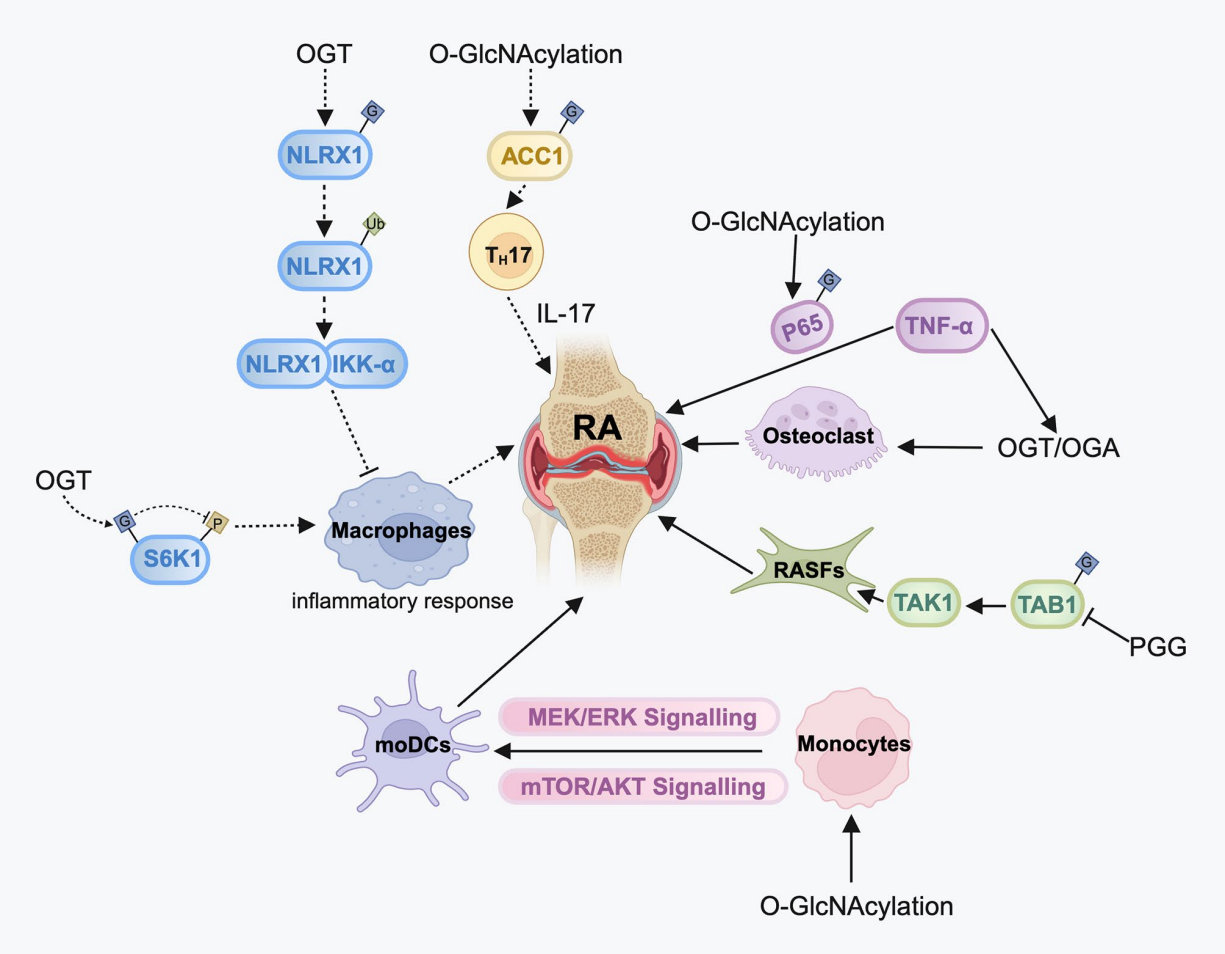
The role of O-GlcNAcylation modification in RA Rheumatoid arthritis (RA) is a chronic inflammatory joint disease associated with loss of cartilage, bone damage, and disability. O-GlcNAcylation may affect the progression of RA by targeting proteins or regulating signaling pathways. Dotted arrows represent a possible but not yet established effect

The pro-inflammatory function of Th17 cells, which originate from CD4^+^ Th cells, is enhanced in RA. Interleukin-17 A (IL-17 A) is the major cytokine that mediates the function of Th17 cells, and it plays a pivotal role in promoting inflammatory reactions [93]. Recent studies have demonstrated the critical role of O-GlcNAcylation nutrient sensing in regulating Th17 cell function. Excessive O-GlcNAcylation can lead to an increase in acetyl-CoA carboxylase 1 (ACC1) O-GlcNAcylation and alterations in the lipidome. These changes in lipid expression activate RORγt, leading to an increase in IL-17 production (Fig. 4) [94]. The above studies hint that regulation of O-GlcNAcylation metabolism in these cells may have clinical applications. However, further research is required to determine whether O-GlcNAcylation directly regulates RA and its underlying mechanisms through the above cells.

### Osteoarthritis (OA)

O-GlcNAcylation is known to play a crucial role in regulating various cellular processes during embryonic development and during immune responses [79]. Nonetheless, the impact of O-GlcNAcylation on inflammation is still not clear. OA is a prevalent and chronic joint disease that causes progressive cartilage degradation, subchondral bone remodeling, bone marrow lesions, meniscal damage, and synovitis [95]. The classical view of the pathogenesis of OA is that subchondral sclerosis is associated with age-related joint degeneration. Notably, clinical investigations on OA patients and research using animal models suggest that there is a connection between abnormal glycosylation and the onset and advancement of OA [96]. An analysis of gene expression data revealed that glycosyltransferase expression was affected by pro-inflammatory cytokines, potentially resulting in protein degradation and an acceleration of OA pathology [97].

**Fig. 5:**
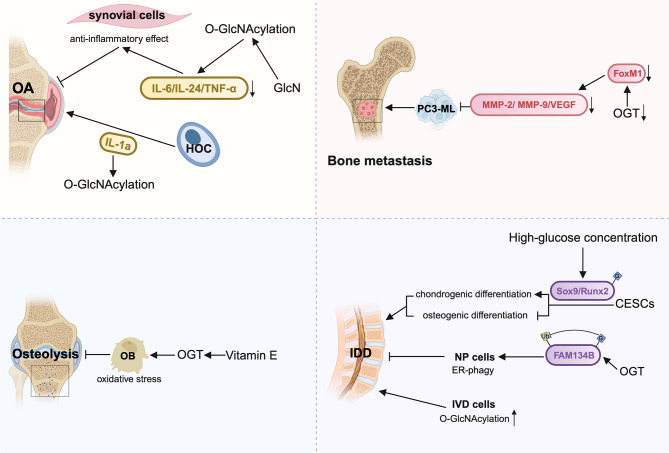
The role of O-GlcNAcylation modification in other bone-related diseases O-GlcNAcylation also plays a role in other bone-related diseases, including OA (Osteoarthritis), osteolysis, bone metastasis and IDD (Intervertebral disc degeneration). Generally, aberrant O-GlcNAcylation modifications on diverse substrates promote or suppress bone-related disease progression

O-GlcNAcylation levels are also known to change significantly during the progression of OA. Glucosamine (GlcN) is a naturally occurring amino monosaccharide widely used as a dietary supplement for OA [98–103]. Someya et al. reported that GlcN exerts an anti-inflammatory effect on synovial cells in OA by suppressing the gene expression of multiple pro-inflammatory cytokines (including IL-6, IL-24, and TNF-α) via O-GlcNAc modification. Thus, GlcN exhibits a protective effect on OA [104]. Interleukin-1 (Il-1), a multifunctional monokine, plays a significant role in the destruction of articular cartilage in degenerative arthropathies [105]. Interestingly, research has shown that the concentration of IL-1a is also increased in OA joints. Moreover, IL-1a induced an accumulation of O-GlcNAcylation in cultured human OA chondrocytes (HOC), thus providing evidence for a link between OA and O-GlcNAc levels (Fig. 5) [106].

### Intervertebral disc degeneration (IDD)

IDD is a common finding on spine imaging, and it becomes more prevalent with age [107]. IDD is characterized by the increased secretion of pro-inflammatory cytokines, including TNF, IL-1α, IL-1β, IL-6, and IL-17, from intervertebral disc (IVD) cells. These cytokines facilitate extracellular matrix degradation, chemokine production, and changes in IDD cell phenotype [108]. Recent studies provide evidence of a direct interaction between OGT and FAM134B protein, resulting in the stabilization of FAM134B via inhibition of ubiquitin-mediated degradation. In addition, this interaction improves the adaptive capability of nucleus pulposus (NP) cells and retards IDD progression by modulating FAM134B-mediated ER-phagy [109]. However, a study on human degenerated intervertebral discs revealed that disc degeneration was associated with increased O-GlcNAcylation of nuclear and cytoplasmic proteins in IVD cells [110]. Diabetes is known to exacerbate disc degeneration by increasing apoptosis and senescence in nucleus pulposus cells [111]. Further study claimed that glucose-induced O-GlcNAcylation of Sox9 and Runx2 altered the fate of chondro-osteogenic differentiation of cartilage endplate stem cells (CESCs), leading to faster degeneration of the intervertebral disc (Fig. 5) [112]. The different reported outcomes of O-GlcNAcylation modification in IDD demonstrate the complexity of this pathway and suggest the importance of additional research.

### Osteolysis

Osteolysis is the loss of bone tissue due to a pathological process [113]. The main mechanism of osteolysis involves an immunologic response to particulate debris, leading to progressive bone loss and implant loosening [114]. To date, an osteolysis diagnosis remains challenging, and the treatments are considered controversial. Massaccesi’s group reported that Vitamin E significantly increased levels of OGT, improving the ability of osteoblasts to respond to oxidative stress and ultimately reducing osteolysis caused by oxidation (Fig. 5) [115]. This study provides new insights into preventing osteolysis and improving the longevity of total joint replacements.

### Bone metastasis

Bone is one of the most common sites targeted in advanced solid tumors, and cancer cells undergo complex biochemical, morphological, pathophysiological, and genetic changes in order to colonize bone sites [116, 117]. Unfortunately, once cancer has spread to the bone, it is rarely curable, and bone cancer is associated with pain, increased risk of fracture, and hypercalcemia [118]. It has been observed that higher levels of O-GlcNAcylation and OGT in cancer patients are associated with cancer progression and a poor prognosis. By reducing OGT levels, the expression level and angiogenic potential of invasion and angiogenesis effectors (MMP-2, MMP-9, and VEGF) in PC3-ML cells can be inhibited, and this process is dependent on increased degradation of FoxM1 [119]. It is important to note that this mechanism could inhibit prostate cancer metastasis to bone (Fig. 5). This discovery sheds light on the significance of OGT-mediated O-GlcNAcylation in bone metastasis. Moreover, it suggests that OGT may be a novel therapeutic target for treating tumor-associated bone metastasis.

## The promising role of O-GlcNAcylation in the treatment of bone diseases

Since the discovery of O-GlcNAcylation in the early 1980s, several studies have addressed the role of protein O-GlcNAcylation in various human diseases. Although we have explored some of its important mechanistic details, research on the treatment of bone diseases through modulation of O-GlcNAcylation is limited. Nonetheless, O-GlcNAcylation is expected to become a new molecular marker and target for the treatment of bone disease. There are currently two main strategies for regulating the O-GlcNAcylation pathway to achieve therapeutic effects [120]. The first strategy involves the targeting of O-GlcNAcylation modifying enzymes, OGT and OGA. The second strategy is more targeted, and it involves changing the O-GlcNAcylation moieties on specific target proteins. This targeted approach has obvious advantages.

**Fig. 6:**
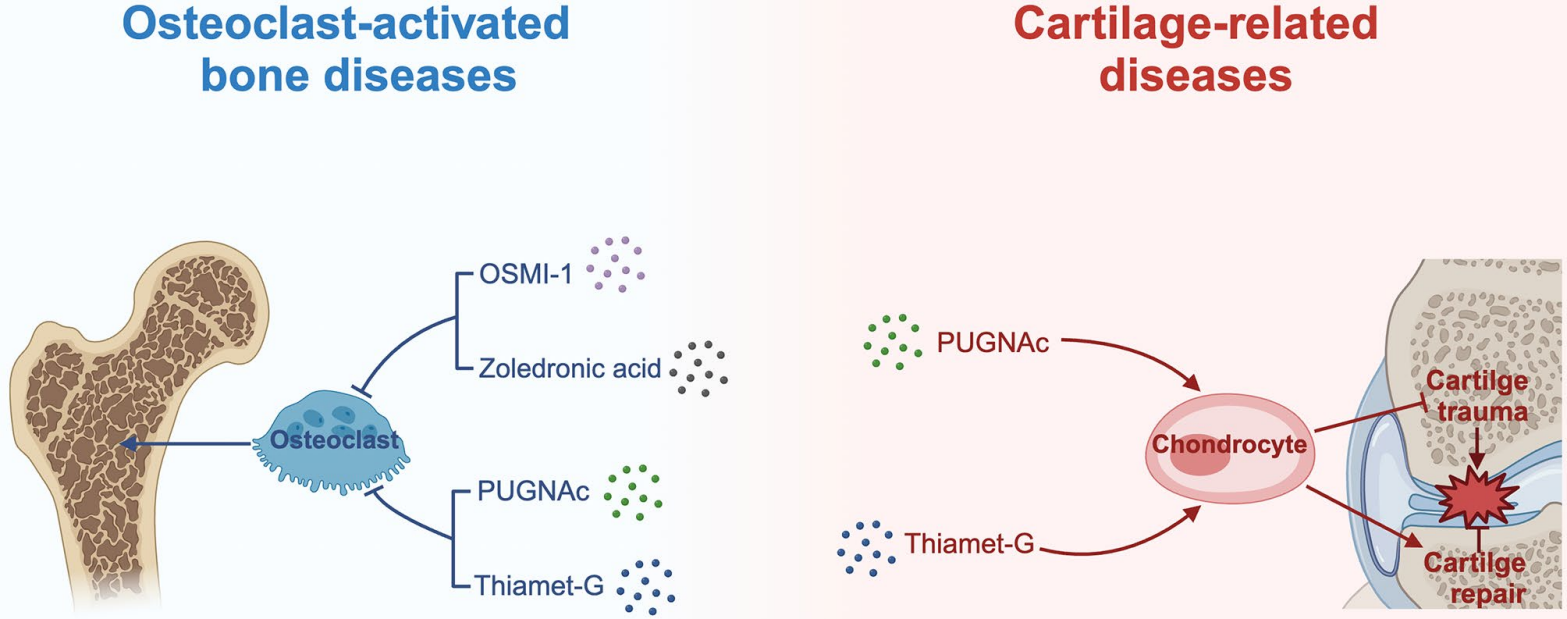
Strategy for targeting O-GlcNAcylation in the treatment of bone diseases OSMI-1, PUGNAc, and Thiamet-G mediated O-GlcNacylation can inhibit osteoclast differentiation and may have potential use in the future for preventing or treating osteoclast-activated bone diseases. PUGNAc and Thiamet-G administration may offer new avenues for studying cartilage formation, metabolism, and repair, by acting on chondrocytes

Previous reports have described the effects of several small molecule inhibitors of OGT and OGA on disease states. OSMI-1 is a cell-permeable, small-molecule inhibitor of OGT that inhibits O-GlcNAcylation [121]. Zoledronic acid is a potent amino bisphosphonate that is commonly used worldwide for the treatment of primary or secondary osteoporosis and low bone mass [122]. An investigation into the mechanism by which OSMI-1 interferes with osteoclastogenesis revealed that the effects of OSMI-1 and zoledronic acid on osteoclast differentiation were synergistic [51]. Interestingly, PUGNAc and Thiamet-G, commonly used in vitro and in vivo inhibitors of OGA, have also demonstrated inhibition of osteoclast differentiation [123–126]. This result can be explained by considering the complex off-target effects of inhibitors in different cell environments under different treatment regimens. Similarly, according to a study by Riegger’s group, PUGNAc exhibits both chondroprotective effects and chondroanabolic effects after cartilage trauma [127] (Fig. 6). Thiamet-G has also been shown to evoke both chondrocyte differentiation and matrix remodeling by increasing the presence of different matrix metalloproteinases (MMPs). Despite the promising pre-clinical data from the above findings, there are still many issues that need to be addressed. Inhibitors of OGT and OGA have a broad specificity and may alter the O-GlcNAcylation of thousands of proteins. Unspecified alterations in the patterns of O-GlcNAcylation on affected proteins could lead to severe side effects or new conditions. In addition, their high toxicity, low efficiency, low water solubility and cell permeability make in vivo studies difficult [128, 129].

To address these problems, novel approaches for the selective protein regulation of O-GlcNAc are required. The development of liquid chromatography-mass spectrometry (LC-MS) has enabled accurate and large-scale prediction of O-GlcNAcylation sites in specific proteins. Retention and slow release of small molecules drugs at the lesion site by constructing a drug delivery system improves the targeting ability of O-GlcNAcylation and reduces systemic side effects. Yang et al. reported that nanocarriers allow selective delivery of OGT inhibitors or other compounds to target cells, thereby improving the efficiency of O-GlcNAcylation targeting [129]. In addition, Zhu et al. developed a notable strategy that involves using short peptides containing glycosylation sites to competitively inhibit glycosylation in a specific protein [130]. These strategies should provide future opportunities for designing targeted drug therapies. However, the relevant research is still evolving, and there is a long way to go to take these developments through to clinical applications.

## Conclusions

Researchers have extensively studied the mechanism of protein O-GlcNAcylation over the past 30 years. O-GlcNAcylation is an essential component in the integration of metabolism with transcription, translation, proteostasis, and signaling (among others). Although the significance of O-GlcNAcylation in bone is increasingly recognized, our understanding of the molecular mechanisms by which O-GlcNAcylation regulates signaling events in bone and how this alters bone pathophysiology is still superficial. Hence, these are essential areas for future research. Moreover, through the development and increased accessibility of tools for detecting O-GlcNAcylation, especially LC-MS, our understanding of O-GlcNAcylation in the proteome is constantly being increased. With a comprehensive knowledge of O-GlcNAcylation in the proteome, we will be better able to understand its complex role in biological processes and to develop its potential as a therapeutic target for various types of bone diseases.

## Data Availability

Not applicable.

## Abbreviations

O-GlcNAcylation: O-linked N-acetylglucosamine protein modification
PTM: post translational modification
GlcNAc: N-acetylglucosamine
HBP: hexosamine biosynthetic pathway
UDP-GlcNAc: UDP N-acetyl-glucosamine
HK: hexokinase
GPI: glucose 6-phosphate isomerase
GFAT: Glutamine fructose-6-phosphate amidotransferase
GlcN-6P: glucosamine 6-phosphate
GNPNAT, EMeg32: glucosamine phosphate N-acetyltransferase
GlcNAc-6P: N-acetylglucosamine-6-phosphate
PGM3/AGM1: GlcNAc phosphomutase
GlcNAc-1P: N-acetylglucosamine-1-phosphate
UAP/AGX1: pyrophosphorylase
TPR: tetratricopeptide repeats
PPIs: protein protein interactions
GH84: 84 glycoside hydrolase
pHAT: pseudo histone acetyltransferase domain
BMSCs: bone marrow mesenchymal stromal cells
NSCs: neural stem cells
HSCs: hematopoietic stem cells
BMP: bone morphogenetic protein
Hh: hedgehog
Runx2: Runt related transcription factor 2
ALP: alkaline phosphatase
MAPK: mitogen activated protein kinase
ERK: extracellular signal regulated kinase
JNK: Jun N-terminal protein kinases
NF-κB: nuclear factor kappaB; nuclear factor of activated T cells c1
IL-6: Interleukin 6
FAS: fatty acid synthase
LD: lipid droplet
GlcN: glucosamine
RA: Rheumatoid arthritis
TNFα: Tumor necrosis factor alpha
IL-1β: Interleukin 1β
TAB1: transforming growth factor beta activated kinase 1-binding protein 1
TAK1: TGFβ activated kinase 1
RASFs: RA synovial fibroblasts
DCs: Dendritic cells
moDCs: Monocyte derived dendritic cells
M1: classically activated macrophages
M2: alternatively activated macrophages
S6K1: S6 kinase beta 1
NLRX1: NOD like receptor (NLR)X1
IL-17 A: Interleukin 17 A
ACC1: acetyl-CoA carboxylase 1
OA: Osteoarthritis
Il-1: Interleukin-1
HOC: human OA chondrocytes
IDD: Intervertebral disc degeneration
CESCs: cartilage endplate stem cells
VEGF: vascular endothelial derived growth factor
LC-MS: liquid chromatography-mass spectrometry
